# Knowledge and Utilization of Information Technology Among Health Care Professionals and Students in Ile-Ife, Nigeria: A Case Study of a University Teaching Hospital

**DOI:** 10.2196/jmir.6.4.e45

**Published:** 2004-12-17

**Authors:** Ibrahim S Bello, Fatiu A Arogundade, Abubakr A Sanusi, Ikechi T Ezeoma, Emmanuel A Abioye-Kuteyi, Adewale Akinsola

**Affiliations:** ^2^Department of MedicineObafemi Awolowo University Teaching Hospitals ComplexIle-IfeNigeria; ^1^Department of General Medical PracticeObafemi Awolowo University Teaching Hospitals ComplexIle-IfeNigeria

**Keywords:** Information management, information retrieval, information technology, health care providers, students, Nigeria

## Abstract

**Background:**

The computer revolution and Information Technology (IT) have transformed modern health care systems in the areas of communication, teaching, storage and retrieval of medical information. These developments have positively impacted patient management and the training and retraining of healthcare providers. Little information is available on the level of training and utilization of IT among health care professionals in developing countries.

**Objectives:**

To assess the knowledge and utilization pattern of information technology among health care professionals and medical students in a university teaching hospital in Nigeria.

**Methods:**

Self-structured pretested questionnaires that probe into the knowledge, attitudes and utilization of computers and IT were administered to a randomly selected group of 180 health care professionals and medical students. Descriptive statistics on their knowledge, attitude and utilization patterns were calculated.

**Results:**

A total of 148 participants (82%) responded, which included 60 medical students, 41 medical doctors and 47 health records staff. Their ages ranged between 22 and 54 years. Eighty respondents (54%) reportedly had received some form of computer training while the remaining 68 (46%) had no training. Only 39 respondents (26%) owned a computer while the remaining 109 (74%) had no computer. In spite of this a total of 28 respondents (18.9%) demonstrated a good knowledge of computers while 87 (58.8%) had average knowledge. Only 33 (22.3%) showed poor knowledge. Fifty-nine respondents (39.9%) demonstrated a good attitude and good utilization habits, while in 50 respondents (33.8%) attitude and utilization habits were average and in 39 (26.4%) they were poor. While 25% of students and 27% of doctors had good computer knowledge (P=.006), only 4.3% of the records officers demonstrated a good knowledge. Forty percent of the medical students, 54% of the doctors and 27.7% of the health records officers showed good utilization habits and attitudes (P=.01)

**Conclusion:**

Only 26% of the respondents possess a computer, and only a small percentage of the respondents demonstrated good knowledge of computers and IT, hence the suboptimal utilization pattern. The fact that the health records officers by virtue of their profession had better training opportunities did not translate into better knowledge and utilization habits, hence the need for a more structured training, one which would form part of the curriculum. This would likely have more impact on the target population than ad hoc arrangements.

## Introduction

Since the development of the computer and the evolution of the Internet, Information Technology (IT) has had a positive impact on health care delivery systems worldwide, particularly in the areas of disease control, diagnosis, patient management and teaching [1-3].

While the use of CD-ROM and interactive software packages have greatly contributed to dissemination of information among health care professionals, its use is still very limited in developing countries in Africa [4,5]. The computer and IT offer the physician the ability to store and retrieve patient clinical and sociodemographic information, laboratory results and preparation of referral notes. It also aids the preparation of discharge summaries, clinic letters and financial statements of the hospital [6], as well as delivery of laboratory results [6].

The Internet provides opportunities to retrieve up-to-date information on different aspects of diseases, interact with colleagues via videoconferencing, and enhance communication amongst colleagues in different continents. Free access to Medline, medical journals, textbooks and the latest information on breakthroughs in medicine also encourages learning and research [7].

With a population of approximately 120 million people, Nigeria is the most populous country in Africa. Knowledge of computers and IT had remained poor in Nigeria. Ajuwon and colleagues [8] looked at computer and Internet use by first year clinical and nursing students at the University College Hospital in Ibadan, Nigeria and found that while about 60% had used the Internet and email, only 42.6% of them could use a computer. Ogunyade and Oyibo [4] at the College of Medicine, University of Lagos established that 52% of the 250 medical students in the study were aware of Medline on CD-ROM while only 24% had utilized it. Odusanya and Bamgbala [5] in the same institution found that 80% of the medical and dental students in their final year had used the computer; however, the use of software applications was poor, with computer games being the most frequently used (19%) followed by word processing software (18%). The Internet and email were used by 58%, but only 23% had used the Internet for medical research. All these studies concluded that that utilization of computers and IT was poor amongst Nigerian students. In sharp contrast to these findings, a Malaysian study by Nurjahan et al [9] found that 94.3% of the studied population had used a computer either in the university or at home. Of that group, 55% had adequate word processing skills, 78% had used email and 67% had surfed the Internet.

Little is known about the perception and utilization patterns of students, and to our knowledge there are no published reports on the knowledge and utilization patterns of IT among health care professionals in Nigeria.

In 2001, Edworthy reviewed the applications of telemedicine and felt that it may in fact have a more profound impact on developing countries than on developed ones. He noted that even in very remote and relatively underdeveloped communities such as the satellite stations in Uzbekistan, Cambodia, and Kosovo, low bandwidth Internet reached into the most remote areas, despite their unstable political climate and poor socioeconomic environments [2].

**Figure 1:**
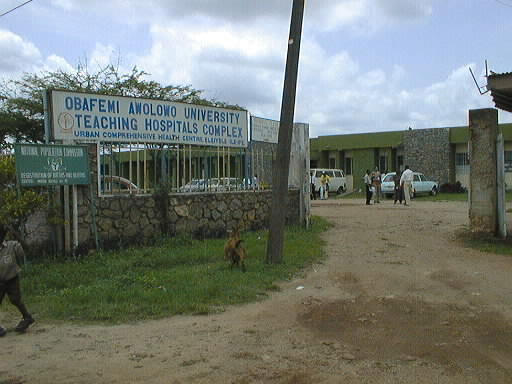
An annex of Obafemi Awolowo University Teaching Hospital Complex in Ile-Ife, Nigeria (Comprehensive Health Center, Eleyele) (Photo: Anja Mursu)

**Figure 2:**
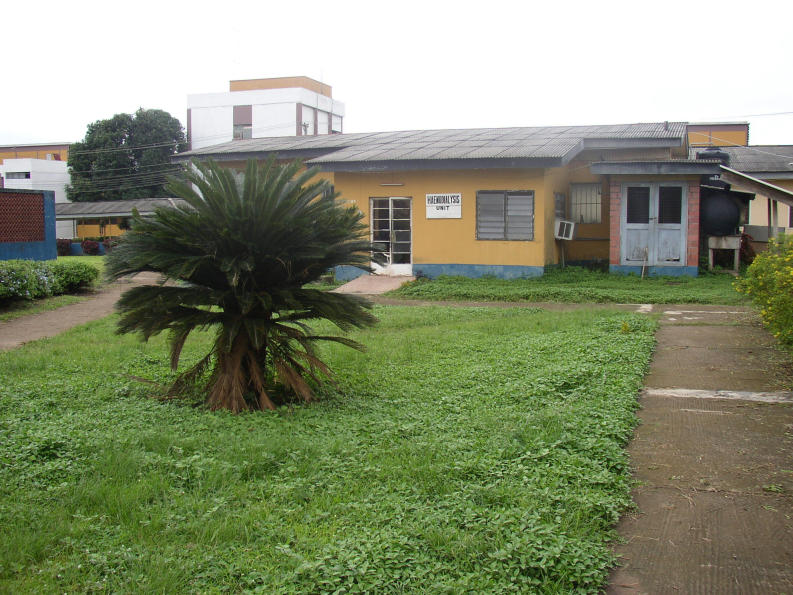
Renal Center-Haemodialysis Unit at the hospital (Photo: Fatiu Arogundade)

An information-proficient workforce that is computer literate, trained in information management skills and motivated to use the well-designed clinical systems would be necessary in a tertiary institution particularly in a developing country such as Nigeria. Clinical informatics aims to improve patient care by the intelligent application of technology and hopes to increase the effectiveness and efficiency of care, as well as patient safety [10,11]. Informatics can fulfil its promises in developing countries only if health care professionals are trained in basic computing skills and IT. Designing such training will necessitate an assessment of baseline knowledge and the utilization patterns of all personnel involved in health care delivery which is the major thrust of this survey.

The aim of this study is to assess the knowledge and utilization patterns of IT among health care professionals and students in Nigeria using the Obafemi Awolowo University Teaching Hospital in Ile-Ife as a case study.

**Figure 3:**
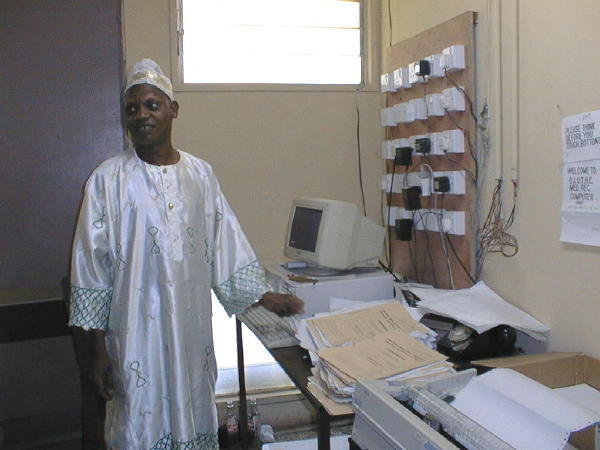
A senior health records officer in the hospital's coding room at the Obafemi Awolowo University Teaching Hospital, Ile-Ife, Nigeria (Photo: Anja Mursu)

## Methods

The survey was conducted at the Obafemi Awolowo University Teaching Hospitals Complex (OAUTHC) in Ile-Ife, Nigeria. OAUTHC is one of the first-generation teaching hospitals established by the Nigerian government to deliver quality health care to its people, and was, until very recently, the only teaching hospital in Osun State, drawing patients from the whole of Ondo, Ekiti and parts of Oyo and Kwara states, which include a predominantly Yoruba ethnic population of about 20 million.

Launched in 1977 at the then fledgling University of Ile-Ife (now Obafemi Awolowo University), the hospital complex has grown to encompass 2 major hospital facilities, 1 dental hospital and 3 primary care centers. The major centers include the tertiary referral center in Ile-Ife and the Wesley Guild Hospital at Ilesa, located in a rural setting 30 kilometers from Ile-Ife. OAUTHC has 565 beds, and (in 2003) saw 204669 patients.

A pretested questionnaire that was to be self-administered, and which probed into the knowledge, attitude and utilization of computer and IT, was given to health care professionals and medical students (see Multimedia Appendix). We distributed 180 questionnaires to randomly selected staff which included 3 groups of personnel: medical doctors (n = 60), health record staff (n = 60) and medical students (n = 60).

The randomly selected medical students were in their fourth and fifth years of training at the Obafemi Awolowo University. The preclinical class was not studied because the students were located at another campus.

Respondents' names were not elicited in the questionnaire in order to enhance participation and reduce respondent bias.

The first section of the questionnaire (see Multimedia Appendix) sought sociodemographic information of the respondents. Computer knowledge was assessed by analyzing responses to a set of 19 questions (questions 7 to 25) while another set of 16 questions (questions 26 to 40) was used to determine attitude and utilization patterns. Continuous scores from these categories were converted into an ordinal “good-fair-poor” scale. Participants who scored greater than 80% on the knowledge questions were categorized as having “good” computer knowledge. Those with scores between 60% and 79% were determined to have “fair knowledge”, while individuals with scores less than 60% were categorized as having “poor knowledge”.

In the “attitude and utilization” categories, total scores above 60% were categorized as “good”, while scores ranging between 50 and 59% were rated as “fair”, and those with scores less than 50% were considered to have “poor” attitude and utilization skills.

### Statistical Analysis

Data analysis was done using SPSS package version 10 and Computerized Programme for Epidemiological Analysis. Comparison of knowledge and utilization patterns in different groups was done using an independent Student's t-test. Pearson correlation was used to evaluate an association between knowledge and utilization patterns. Chi-square and Fisher's exact tests were used when appropriate to find any associations between the categorical variables.

## Results

### Sociodemographic Characteristics of Respondents

Out of 180 who received questionnaires, 148 (82 %) responded, including 60 medical students, 41 medical doctors and 47 health records staff. Their ages ranged between 22 and 54 years. The majority of respondents (120) were males, while the remaining 28 were females (Table 1).

**Table 1:** Sociodemographic data of respondents and type of computer training

		**Students** **(n = 60)**	**Doctors** **(n = 41)**	**Health Records Officers (n =47)**
**Age Range in years (Mean ± SD)**		22-32 (24.7 ± 2.29)	26-54 (33.02 ± 6.46)	20-51 (35.83 ± 8.41)
**Gender**	Male	52 (86.7%)	39 (95.1%)	29 (61.7%)
	Female	8 (13.3%)	2 (4.9%)	18 (38.3%)
**Marital Status**	Single	58 (96.7%%)	22 (53.7%)	10 (21.3%)
	Married	2 (3.3%)	17 (41.5%)	35 (74.4%)
	Divorced	-	1 (2.4%)	-
	Widowed	-	1 (2.4%)	-
	Separated	-	-	2(4.3%)

### Computer Possession and Training Received

Eighty respondents (54%) had received some form of computer training, while the remaining 68 (46%) had no training. Only 39 respondents (26%) owned a computer while the remaining 109 (74%) had no computer (Table 2).

**Table 2:** Previous computer training received by the respondents

		**Students** **(n = 60)**	**Doctors** **(n = 41)**	**Health Records Officers** **(n = 47)**
**Number of respondents with computer training**		21 (35%)	22 (53.7%)	37 (78.9%)
**Computer possession**	Yes	17 (28.3%)	19 (46.3%)	3 (6.4%)
	No	43 (71.7%)	22 (53.6%)	44 (93.6%)
**Knowledge**	Good	15 (25%)	11 (27%)	2 (4.3%)
	Fair (Average)	31 (51.7)	25 (61%)	31 (66%)
	Poor	14 (23.3%)	5 (12%)	14 (29.8%)
**Attitude and utilization**	Good	24 (40%)	22 (54%)	13 (27.7%)
	Fair (Average)	25 (41.7%)	11 (27%)	14 (29.8%)
	Poor	11 (18.3%)	8 (19%)	20 (42.6%)

**Table 3:** Computer possession, knowledge and utilization patterns among respondents

		**Students** **(n = 60)**	**Doctors** **(n = 41)**	**Health Records Officers** **(n = 47)**
**Previous computer training received**	Short course	16 (26.7%)	13 (31.7%)	19 (40.4%)
	Certificate course	4 (6.7%)	5 (12.2%)	5 (10.6%)
	Diploma course	1 (1.7%)	4 (9.8%)	9 (19.1%)
	Degree course	-	-	4 (8.5%)
	No training	39 (65%)	19 (46.3%)	10 (21.3%)

### Knowledge and Utilization Pattern of Respondents

A total of 28 respondents (18.9%) demonstrated a good knowledge of computers while 87 (58.8%) of them had an average knowledge. Only 33 (22.3%) showed poor knowledge. Fifty-nine respondents (39.9%) had good attitude and utilization habits, while 50 (33.8%) showed average or fair habits and 39 (26.4%) exhibited poor attitude and utilization habits.

Only 4.3% of the records officers demonstrated good knowledge, compared to 25% of students and 27% of doctors (*P*=.006) (Table 3). The knowledge scores were not significantly different between medical students and health records officers (*P*=.06), or between the medical students and the doctors (*P*=.374). However, the doctors' scores were significantly higher than those of the health records officers (*P*=.001).

Forty percent of medical students, 54% of the doctors and 27.7% of the health records officers scored “good” in the attitudes and utilization category (*P*=0.01). The medical students' attitude and utilization scores were significantly higher (*P*=.008) than those of the health records officers, as were the doctors' scores (*P*=.001). However, the difference between the doctors' and medical students' attitude and utilization scores were not significant (*P*=.10).

The detailed data for the three different groups are further elaborated below.

### Computer Training Received, Knowledge and Utilization Pattern in the 3 Study Groups

#### Medical Students

The age of the medical students ranged from 22 to 32 years (mean = 24.7; SD = 2.29). Thirty (50%) of them were in the fifth year of their course, 28 (46.7%) were in the fourth year and only 2 were in the sixth (final) year. Fifty-two (86.7%) of them were males while the remaining 8 (13.3%) were females. With regard to marital status, 58 (96.7%) were single and 2 (3.3%) were married. Despite the fact that 35% of the students had some training in computers and IT, only 15 (25%) demonstrated good knowledge in this field, 31 (51.7%) had a fair knowledge, and in the remaining 14 (23.3%) knowledge base was poor. Of the 35% who had received training, 16 (26.6%) had informal training, 5 (8.3%) attended a certificate course and only 1(1.6%) had a diploma. The training was not found to influence their computer/IT knowledge (*P*=.8). Forty percent of them had "good" attitude and utilization habits while attitude and utilization habits were "fair" in 41.7% and "poor" in 18.3% of the students (Table 3).

#### Medical Doctors

Among the doctors recruited, age ranged from 26 to 54 years (mean = 33.02; SD = 6.46). The majority were males (39; 95.1%), while only 2 females (4.9%) responded. Fifty-eight percent were married while the remaining 41.5% were single. While 46.3% had no training, 53.7% had varying degrees of exposure, out of which 31.7% had informal training, 12.2% had taken a certificate course and the remaining 9.8% had taken a diploma course. Despite this training only 27% of the doctors had good knowledge scores, while 61% showed fair knowledge and 12% had poor knowledge. With regard to attitude and utilization habits, 54% of the doctors had good scores, 27% had fair scores and 19% showed poor attitude and utilization habits. Marital status, number of children, gender and computer training were not found to influence significantly knowledge and utilization habits (Table 3).

#### Health Records Officers

The age of the health records staff ranged from 20 to 51 years (Mean = 35.83; SD = 8.41). There were 18 (38.3%) females and 29 (61.7%) males. Thirty five (74.4%) were married with children while 10 (21.3%) were single and 2 (4.3%) were separated. Despite the fact that 78.7% had received some training, only 4.3% demonstrated good knowledge of computers and IT. Sixty-six percent had a fair knowledge and 29.7% had poor knowledge. Also only 27.7% showed good utilization habits, while in 29.8% they were fair and in 46.6% they were poor. Rank, social obligations, age and gender were not found to significantly influence knowledge, utilization patterns and attitudes (Table 3).

## Discussion

In this study we found that computer possession and utilization among health care professionals and students in a major university teaching hospital in Nigeria were low. Only 26% of respondents owned a computer and 18.9% and 39.9% had good knowledge and utilization habits respectively. This is similar to the findings in other parts of Nigeria. Ajuwon [8] found that only 42.6% of medical and nursing students could use a computer while about 60% had used the Internet. Ogunyade and Oyibo [4] discovered that 52% of the 250 students studied were aware of Medline on CD-ROM while only 24% had used it. In sharp contrast to our findings, Odusanya and Bamgbala [5] in Lagos reported that 80% of their final year medical and dental students had used the computer, but the use of software applications was very poor (19%).

Among our medical student population, 25% demonstrated good knowledge while 40% showed good utilization patterns, which is in agreement with the findings of other researchers in Nigeria, but significantly lower than the figures from Malaysia [9], Jeddah (Saudi Arabia) [12], Glasgow (UK) [13] and Oulu in Finland [14]. Lack of structured training and computer accessibility may have contributed to the poor knowledge and utilization patterns observed. In addition limited access to the Internet and the relatively expensive nature of Internet cafés may also be contributory. Provision of computer laboratories in various departments in our universities where students can have full access to Internet services that are cost free would certainly assist in improving utilization pattern and, hence, the acquisition of knowledge.

Computer possession was found to be higher among doctors when compared with the 2 other groups; this may be because of the perceived need and relative ease of affordability. Unfortunately only 27% of the doctors demonstrated good knowledge and 54% showed good utilization habits. These were not statistically different from those of the students.

Despite the lowest computer possession rate (4.3%), 78.9% of health records personnel had received training on computer use and IT. This was a result of required job training which was usually employer sponsored as opposed to the other 2 groups who sought training for personal and/or professional reasons. Surprisingly, both knowledge and utilization scores were statistically lower among health records officers when compared with the medical students or doctors. This may be related to their level of educational attainment since only a diploma is required to become a health records officer, although many senior officers have higher levels of education including university degrees.

The finding of higher utilization patterns compared with computer knowledge among all the respondents is not surprising as most respondents who do not own computers utilize them by going to Internet cafés where attendants can be found to assist them. This is consistent with the findings of others [5,8,9].

The gains of IT can only be fully harnessed when the majority, if not all, of the staff become knowledgeable and are willing to utilize computers and IT. Such utilization will naturally impact on health information management. The use of Medline, CD-ROMs and interactive software packages would enhance dissemination of medical information, knowledge and teaching among health care professionals. It would also improve health care delivery and collaborative multicenter research, which is still very limited in the developing countries particularly in Africa [4,5].

It has been established that computers and IT can have numerous applications ranging from storage and retrieval of patient clinical and sociodemographic information to patient management, particularly in specialties such as cardiology, neurology, pediatrics, otorhinolaryngology, general practice (family medicine) [15-19] and even in hospital administration [20].

The availability of email, websites, chat rooms, multimedia presentations, and occasional opportunities for communication via Internet phones, videoconferencing and even Internet conferencing have rejuvenated medical education and teaching, patient care, and collegial support [21].

Our student population has increased over the years and even now would likely overwhelm our facilities if it were not for the use of IT enabling lectures, demonstrations and illustrations to be delivered to multitudes of students simultaneously.

The fact that the health records officers by virtue of their profession had better training opportunities did not translate into better knowledge and utilization habits, which raises questions about the style and type of computer and IT training offered. It is our belief that a structured training, which forms part of the curriculum, would likely have more impact on the target population than ad hoc arrangements. The introduction of a structured computer training course, which includes the applicability of IT to medicine, into the curriculum of medical students, health record students, residency and continuous medical education training (CME) programs for all practicing physicians and health workers would certainly assist in ensuring maximal utilization of the innumerable advantages offered by IT.

Medicine is an ever-evolving and information-based discipline, and as such the provision of structured computer and IT training for all members of the health team would equip them with the skills they need to practice up-to-date and evidence-based medicine, which are essential to improving the quality of medical care.

Further research should focus on designing and evaluating computer and IT training for students and staff in developing countries.

## Multimedia Appendix

Information Technology Questionnaire:

## References

[1] Myers Mary R (2003). Telemedicine: an emerging health care technology. Health Care Manag (Frederick).

[2] Edworthy S M (2001). Telemedicine in developing countries. BMJ.

[3] Feliciani Francesco (2003). Medical care from space: Telemedicine. ESA Bull.

[4] Ogunyade Taiwo O, Oyibo Wellington A (2003). Use of CD-ROM MEDLINE by medical students of the College of Medicine, University of Lagos, Nigeria. J Med Internet Res.

[5] Odusanya O O, Bamgbala O A (2002). Computing and information technology skills of final year medical and dental students at the College of Medicine University of Lagos. Niger Postgrad Med J.

[6] Majeed Azeem (2003). Ten ways to improve information technology in the NHS. BMJ.

[7] Turner Jeanine Warisse, Robinson James D, Alaoui Adil, Winchester James, Neustadtl Alan, Levine Betty A, Collmann Jeff, Mun Seong K (2003). Media attitudes vs. use: the contribution of context to the communication environment in telemedicine. Health Care Manage Rev.

[8] Ajuwon Grace Ada (2003). Computer and internet use by first year clinical and nursing students in a Nigerian teaching hospital. BMC Med Inform Decis Mak.

[9] Nurjahan M I, Lim T A, Yeong S W, Foong A L S, Ware J (2002). Utilization of information technology in medical education: a questionnaire survey of students in a Malaysian institution. Med J Malaysia.

[10] Celler Branko G, Lovell Nigel H, Basilakis Jim (2003). Using information technology to improve the management of chronic disease. Med J Aust.

[11] Wickramasinghe Nilmini, Silvers J B (2003). IS/IT the prescription to enable medical group practices attain their goals. Health Care Manag Sci.

[12] Mansor I (2002). Computer skills among medical students: a survey at the King Abdul Aziz University, Jeddah. J Ayub Med Coll.

[13] Jones R B, Navin L M, Barrie J, Hillan E, Kinane D (1991). Computer literacy among medical, nursing, dental and veterinary undergraduates. Med Educ.

[14] Virtanen Jorma I, Nieminen Pentti (2002). Information and communication technology among undergraduate dental students in Finland. Eur J Dent Educ.

[15] James D A, Rowlands D, Mahnovetski R, Channells J, Cutmore T (2003). Internet based ECG medical information system. Australas Phys Eng Sci Med.

[16] Maulden Sarah A (2003). Information technology, the internet, and the future of neurology. Neurologist.

[17] Kim George R, Lehmann Christoph U (2003). The impact of the Internet on pediatric medicine. Paediatr Drugs.

[18] Holtel Michael R, Burgess Lawrence P A (2002). Telemedicine in otolaryngology. Otolaryngol Clin North Am.

[19] Smith Jason J, Mallard-smith Rebecca J, Beattie Victoria, Beattie David K (2003). Use of information technology in general practice. J R Soc Med.

[20] Moulin T, Retel O, Chavot D (2003). Impact of new information and communication technologies (NTIC) on hospital administration and patient management. Care Network for Diagnosing and Treating Neurologic Emergencies. Sante Publique.

[21] Cooke F J, Holmes A (2000). E-mail consultations in international health. Lancet.

