# Supplementary material for: Knowledge and Utilization of Information Technology Among Health Care Professionals and Students in Ile-Ife, Nigeria: A Case Study of a University Teaching Hospital
Source: J Med Internet Res. 2004 Dec 17;6(4):e45. doi: 10.2196/jmir.6.4.e45 (PMC1550621; doi:10.2196/jmir.6.4.e45)
Supplement: Supplementary file 1 [file jmir_v6i4e45_app1.pdf]

APPENDIX I

INFORMATION TECHNOLOGY QUESTIONNAIRE

The following questions are designed to access the attitude, knowledge and utility of information technology among Physicians in a Nigerian Teaching Hospital. The result of the study will assist relevant authorities in designing relevant programme of intervention for this group.

Participation in this study is voluntary and your confidentiality is assured. Your name is not required in this project.

Thank you for your cooperation.

SECTION 1: DEMOGRAPHIC

1. What is your age? …………………………………………... Years

2. What is your sex?
   Female…………1
   Male……………2

3. What is your current marital status?
   Single…………..1
   Married………....2
   Widowed……….3
   Divorced………..4
   Separated…….…5

4. Do you have any children?
   Yes……………..1
   No……………...2

5. What is your religion?
   Christianity…….1
   Islam…………...2
   Others…………..3
   None……………4

6. Rank
   Consultant……...1
   Senior registrar…2
   Registrar………..3
   Sen. Hous Off….4
   House Officer…..5
   Gen. pract……....6

SECTION 2: KNOWLEDGE OF COMPUTERS

Choose True (T) or False (F)

7. A compact disc is hardware — True…. False…. .
8. The CPU is hardware — True……False…..
9. A File server has a very large hard disc — True…... False….
10. Communication between PC Made by different manufacturers is impossible — True….False……

11. Only data base network can be assessed using a network — True……False…..

12. Digital sound and video can be communicated over a network — True…… false…..

13. A network is a group of computer connected by satellite — True…… false…..

14. Networks allows the diff PCs to access the same files — True…… False.....

15. The global communication network is called the Internet — True…… False….

16. The Internet was originally developed by the military — True…… False….

17. Companies with email may no longer need to use the postal system — True…….False….

18. Message can be sent by email to every PC network instantly — True…….False…..

19. WWW stands for World Wide Web — True…….False…..

20. EMail is short for electronic mail — True…….False……
..

21. The floppy disc has a larger capacity than the compact disc — True…….False…..

22. Ram refers to read only memory — True…….False…..

23. A modem allows computers to communicate using telephone line — True……..False….

24. The modem is short for modular demodulator — True……..False…...

25. People who work at home often communicate with their office using the modem — True……...False.........

SECTION 3: ATTITUDES

25. Have you had any formal training in Computers?
Yes……………1
No……………..2

27.	If yes, indicate whether	Short course……1<br>Certificate……...2<br>Diploma………..3<br>Degree…………4
28.	Do you have a personal computer?	Yes……………..1<br>No……………...2
30.	If yes, which type?	Desktop………...1<br>Laptop…………2<br>Both…………….3
31.	Do you have access to interactive CD ROMS on your field of specialty	Yes……………..1<br>No……………...2
32.	Do you enjoy working on the computer?	Yes……………..1<br>No……………...2<br>Not sure………...3
33.	Do you think information technology has a role in your profession?	Yes……………..1<br>No……………...2<br>Not sure………...3

SECTION 4: UTILIZATION

34.	Do you have an email address?	Yes……………..1<br>No……………...2
35.	Do you have access to medical journal/ organization subscription online?	Yes…………..…1<br>No…………..….2
36.	What do you use the Internet for ?	None……………1<br>Mail…………….2<br>Research……….3<br>Film/News……..4<br>Others (mention).5
37.	How often do you access the Internet?	None……………1<br>Once/ week…….2<br>2-3/ week………3<br>4 and more……..4
38.	How many times have you participated in video conferencing?	None……………1<br>Once……………2<br>Twice / more.......3
39.	How many times have you used a power point presentation?	None……………1

Once……………2
Twice/ more……3

40. What is the average hours you spend on the computer per week?

None……………1
1-3 hrs………….2
5-10 hrs………...3
10-14 hrs……….4
Above 14 hrs…...5
